# Serum Folate Correlates with Severity of Guillain-Barré Syndrome and Predicts Disease Progression

**DOI:** 10.1155/2018/5703279

**Published:** 2018-06-14

**Authors:** Yang Gao, Hong-Liang Zhang, Meiying Xin, Dong Wang, Nannan Zheng, Shuang Wang, Jiancheng Xu, Ying Wang, Jie Zhu, Jiachun Feng

**Affiliations:** ^1^Department of Neurology, The First Hospital of Jilin University, Changchun, China; ^2^Department of Life Sciences, The National Natural Science Foundation of China, Beijing, China; ^3^Department of Laboratory Medicine, THE First Hospital of Jilin University, Changchun, China; ^4^Department of Neurobiology, Care Sciences and Society, Karolinska Institutet, Stockholm, Sweden

## Abstract

The aim of this study was to determine the associations between serum folate level and the clinical course and severity of Guillain-Barré syndrome (GBS). We retrospectively enrolled 112 pairs of GBS patients and age- and sex-matched healthy controls with measured serum folate levels. On admission, 21 (18.9%) GBS patients had folate deficiency, of which only two were female patients. Patients with normal folate levels had a shorter disease progression than those with folate deficiency (median progression duration: 6 versus 13 days,* p* < 0.001). Serum folate levels on admission were correlated with progression duration and Medical Research Council (MRC) sum score in the upper limbs at nadir (r = -0.261,* p* = 0.005; r = -0.208,* p* = 0.03) but not with the duration of hospital stay or GBS disability score (*p* > 0.05). Logistic regression analysis revealed that normal folate levels on admission were an independent predictor of faster GBS progression, along with younger age, intact deep sensation, and a lower MRC sum score on admission. These results show that serum folate levels are correlated with the progression duration and severity of GBS. Further studies are required to confirm the potential of folate level as a biomarker for GBS prognosis.

## 1. Introduction

Guillain-Barré syndrome (GBS) is a heterogenous neuropathy characterized by rapid bilateral limb paresis after a triggering immunological event. GBS patients always achieve a maximum deficit within 4 weeks of onset, and most achieve nadir weakness within 2 weeks [1, 2]. The evaluation of GBS is largely based on its clinical presentation, cerebrospinal fluid analysis, and electrophysiology [3]. However, the optimal timing for lumbar puncture and electrophysiological examination is at least 1 and 2 weeks after the onset of weakness, respectively [4, 5]. Thus, biomarkers for the early prediction of clinical course and outcome are urgently needed.

To monitor disease progression and improve the prognosis of GBS, several markers have been identified as predictors for disease severity and outcomes, including serum albumin levels for monitoring treatment response [6], blood glucose levels for short-term prognosis [7], serum sodium levels for outcomes 1 year after onset [8], and plasma cortisol levels for the prediction of respiratory failure [9]. Compared with these markers, folate plays a more fundamental role in polyneuropathy [10]. Therefore, serum folate level could also be a biomarker for disease evaluation and prediction of GBS.

The primary objectives of this study were to determine whether serum folate levels can be a prognostic marker in GBS patients. We investigated the prevalence of folate deficiency in GBS patients and analyzed the association between serum folate levels on admission and GBS disease severity at nadir.

## 2. Methods

### 2.1. Ethical Statements

This retrospective study was approved by the Ethics Committee of the First Hospital of Jilin University and all patients provided signed informed consent. All data were obtained from the electronic medical records at our hospital, and the patients were anonymized.

### 2.2. Subjects

This study included two groups of patients with recorded serum folate levels: (1) GBS patients admitted to the Department of Neurology of the First Hospital of Jilin University between January 2014 and June 2017 and (2) healthy controls who attended the Physical Examination Section of the First Hospital of Jilin University in 2017. Subjects in the control group were matched for sex and age (within a 5-year margin) with GBS patients in a 1:1 ratio. The following GBS patients were excluded: (1) those aged <18 years; (2) those with Miller Fisher syndrome or chronic inflammatory demyelinating polyradiculoneuropathy; and (3) those with a hospital stay of <5 days.

Motor function deficits of the patients were monitored during the study using the Medical Research Council (MRC) sum score [11] ranging from 0 (quadriplegia) to 60 (normal strength) and the GBS disability scale [12] ranging from 0 (healthy) to 6 (dead). The nadir of GBS was defined as the lowest MRC sum score [11]. The progression duration was defined as the number of days between the onset of symptoms and weakness at nadir [13]. A faster disease progression was defined as a peak illness less than or equal to seven days from onset.

### 2.3. Folate Measurement

Serum folate levels were determined by radioimmunoassay (UniCel DxI 800, Beckman Coulter Inc., USA). Folate deficiency was defined as serum folate of <3.5 ng/mL [14]. In normal folate group, high-normal and low-normal levels were defined as >6.0 g/mL and 3.5-6.0 ng/mL, respectively [15].

### 2.4. Statistics

IBM SPSS Statistics for Windows, version 21.0 software (IBM Corp., USA), and GraphPad Prism 7.0 (GraphPad Software Inc., USA) were used for data and used analysis. Case-control matching in SPSS was used to match GBS patients and healthy controls based on gender and age. Shapiro-Wilk tests were used to test the normality of the distributions. Univariate analysis was conducted using Mann–Whitney U tests (for disease duration, MRC score, GBS disability scale, laboratory results, and other variables that were not normally distributed), Kruskal-Wallis tests (for comparison of disease duration among the three groups), Student's t-tests (for age; normally distributed), Pearson Chi-square or Fisher's exact tests (for proportions), and univariable regression analysis (for identification of the predictors of disease progression). Correlations were expressed by Spearman rank correlation coefficients (r). Binary logistic regression was performed with backward selection to identify the independent predictors of disease progression duration. All tests were two-tailed, with a level of significance set to a* p* value of <0.05.

## 3. Results

### 3.1. Serum Folate Levels in GBS Patients and Healthy Controls

A total of 112 pairs of sex- and age-matched GBS patients and healthy subjects were included. Folate deficiency was present in 21 (18.9%) of GBS patients and two (1.8%) of the control group. Serum folate levels in the GBS patients were significantly lower than those in healthy controls (Table 1).

### 3.2. Characteristics of GBS Patients with Folate Deficiency

The 112 GBS patients were divided into two groups depending on whether the serum folic acid level was <3.5 ng/mL. The clinical features of the patients with or without folate deficiency are presented in Table 2 and Table S1. Only two patients (9.5%) in the folate deficiency group were female, while the proportion of female patients without folate deficiency was 50.5% (*p* < 0.001). Patients with normal folate levels had a shorter GBS progression duration than those with folate deficiency (median progression duration: 6 days [IQR 4-11] versus 13 days [7-18], *p* < 0.001).

### 3.3. Association between Serum Folate Level and the Clinical Severity of GBS

As Table 3 shows, there was a significant correlation between serum folic acid level and disease progression duration (r = -0.261, *p* = 0.005). When looking at the correlation by extremities (upper versus lower limbs), the analysis showed a significant correlation between the serum folate level and the MRC score of upper limbs at nadir (r = -0.208, *p* = 0.03) but no correlation between the MRC score or GBS disability score at nadir (r = -0.172, *p* = 0.07; r = 0.114, *p* = 0.23).

### 3.4. Baseline Folate Level Predicts the Length of GBS Progression and Disability at Nadir

Univariate regression analysis was performed to validate the relationship between the serum folate level and GBS disease progression (Table 4). Besides folate deficiency, four other predictors of GBS progression length were also identified: superficial sense deficit, deep sensation deficit, dyspnea on admission, and MRC sum score on admission (all *p* < 0.05). Fasting plasma glucose level, antecedent events, electrophysiology type, history of hypertension or diabetes, and GBS disability score on admission were not significant. The laboratory results of cerebrospinal fluid examinations were not included in the analysis because lumbar punctures were not performed immediately upon admission. To further determine the independent predictors of disease progression, age and gender were also included in the logistic regression analysis model (Table 4). Multivariable analysis showed that GBS patients without folate deficiency were six times more likely to have a faster progression compared to those with folate deficiency (odds ratio [OR], 6.04; 95% confidence interval [CI], 1.69-21.61; *p* = 0.006).

**Figure 1:**
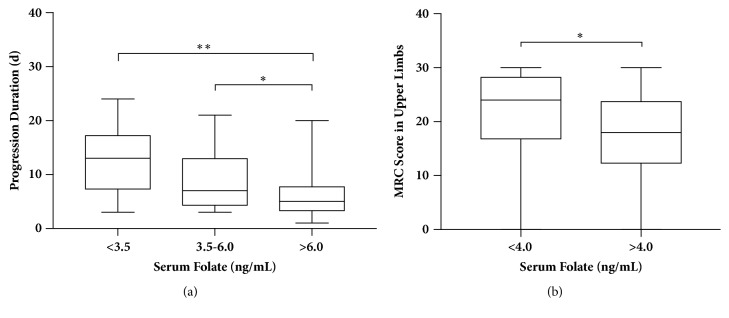
Distribution of progression duration (a) and MRC score in the upper limbs (b), stratified by folate levels. Boxes indicate medians with interquartile ranges (IQRs); *∗* for* p*<0.05 and *∗∗* for* p*<0.01.

To further categorize patients with different folate levels on admission, we divided those with normal levels of folate into two groups. As shown in Figure 1(a), the differences in the medians of progression days between high-normal versus low (*p* = 0.001) or high-normal versus low-normal tertiles (*p* = 0.026) were statistically significant, indicating the value of serum folate level on admission in predicting the length of progression.  Figure 1(b) shows that patients with baseline folate levels of <4.0 ng/mL had a higher MRC score of the upper limbs at nadir compared to the score in those with folate levels of >4.0 ng/mL (*p* = 0.045).

## 4. Discussion

Serum folate levels have been widely measured but rarely studied in GBS patients. We confirmed the occurrence of folate deficiency in GBS patients and identified it as an independent predictor of disease progression duration, together with age, deep sensation, and baseline MRC score. Specifically, higher folate level has been shown to predict a faster disease progression and a worse strength of the upper limbs at nadir.

There are several possible explanations for our finding of insufficient folate levels in GBS patients. Folate deficiency has been shown to diminish human immune functions by affecting T and B cell differentials as well as the proliferation response of lymphocytes [16, 17]. Consequently, it is possible that patients with low folate levels are vulnerable to infections, which may provoke GBS. However, studies have shown that folate supplementation has no effect on infection reduction [18–21]. This suggests a poor association between deficient folate levels and infection susceptibility. On the other hand, GBS as an immune response to infection and infection itself may also cause folate deficiency because these events with rapid cell proliferation could lead to an increased need for folate [22]. We also noticed the impact of gender on folate deficiency in GBS. In general, serum folate level is higher in women than in men [23], which could explain why only two women in our study had folate deficiency. Further, the safety and efficacy of folic acid supplementation in GBS should be carefully evaluated in future studies, as randomized trials have suggested the folic acid may increase the possibility of neoplasms [24–27].

The results of the present study revealed a significant association between folate level on admission and the duration of GBS progression. However, the role of folate deficiency in GBS is unclear. Folate is essential to the peripheral nerves and, and in rare cases, its shortage may cause axonal sensory polyneuropathy [10]. Folate-deficient GBS and folate deficiency neuropathy are both slowly progressive compared to normal folate GBS and thiamine-deficiency neuropathy, respectively. However, this is probably a coincidence because their mechanisms are entirely different. One possible explanation for this association is that deficient folate levels may depress the immune response in GBS and retard the disease progression due to its crucial role in DNA synthesis [28, 29]. Similarly, the potentially concurrent deficiency of other vitamins may also explain the findings [16, 17]; however, this requires further study. Also, the effect of GBS variants cannot be ignored, but our analysis did not prove the roles of antecedent infection and electrophysiology and made GBS forms appear less relevant. Folate level also showed a less strong association with the strength of the upper limbs at nadir; thus, its underlying mechanisms require consideration.

Our study had several limitations. Because this study was retrospective in nature and folate levels were measured only on admission but not at the onset or at nadir, the causation between baseline folate levels and the progression duration require validation. In addition, the plateau phase and recovery phase durations were not available in this study, limiting the investigation of folate's role in the complete clinical course.

## 5. Conclusions

In summary, we demonstrated that serum folate level is an independent marker associated with the duration of GBS progression. The roles of folate in GBS pathogenesis and prognosis need to be explored in prospective studies to monitor dynamic serum folate levels.

## Figures and Tables

**Table 1:** Demographics characteristics and serum folate levels.

Group	GBS (n = 112)	HCs (n = 112)	*p*
Age (year)	52.23 (13.61)	51.16 (12.85)	0.55
Female (N, %)	48 (42.9%)	48 (42.9%)	1.00
Folate (ng/mL)	5.34 (3.98-7.99)	9.46 (5.93-12.78)	< 0.001

HCs, healthy controls.

**Table 2:** Characteristics of GBS patients with and without folate deficiency^a^.

Group	GBS with folate deficiency (n = 21)	GBS without folate deficiency (n = 91)	*p*
**Demographics**			
Age, mean (SD)	53.48 (17.20)	51.95 (12.73)	0.70
Female	9.5% (2/21)	50.5% (46/91)	0.001
**Weakness at nadir, median (IQR)**			
GBS disability score	3 (2-4)	4 (3-4)	0.61
MRC score	48 (28.5-50)	40 (24-48)	0.30
**Disease course, median (IQR)**			
Hospital days	15 (12-19)	13 (11-18)	0.55
Ventilator days (n = 10)	13 (8-13)	27 (14-101)	0.27
Progression duration, d	13 (7-18)	6 (4-11)	0.006

MRC, Medical Research Council.

^a^Data are presented as percentage of patients unless otherwise indicated. All items are shown for 112 patients unless otherwise specified.

**Table 3:** Correlation of serum folate level with the severity of GBS.

Correlation	Spearman r	*p*
Progression duration	-0.261	0.005
Length of stay	0.135	0.18
GBS disability score	0.114	0.23
MRC score at nadir	-0.172	0.07
MRC score in upper limbs	-0.208	0.03
MRC score in lower limbs	-0.118	0.22

MRC, Medical Research Council

**Table 4:** Predictors of a faster GBS progression, defined as reaching nadir weakness in 1 week from onset.

	Univariate analysis		Multivariable analysis	
variables	OR (95% CI)	*p*	OR (95% CI)	*p*
Age	0.97 (0.95, 1.00)	0.07	0.96 (0.93, 0.99)	0.02
Gender (Female vs. male)	1.16 (0.55, 2.47)	0.70	-	-
Superficial sense deficit (no vs. yes)	2.68 (1.24, 5.80)	0.01	-	-
Deep sensation deficit (no vs. yes)	5.13 (1.33, 19.82)	0.02	5.17 (1.16, 23.09)	0.03
Dyspnea on admission (no vs. yes)	0.31 (0.10, 0.91)	0.03	-	-
MRC score on admission	0.96 (0.93, 0.99)	0.005	0.94 (0.90, 0.98)	0.001
Folate deficiency (no vs. yes)	4.19 (1.49, 11.83)	0.007	6.04 (1.69, 21.61)	0.006

OR, odds ratio; CI, confidence interval; MRC, Medical Research Council

## Data Availability

The data generated or analyzed during this study are included in this article and the supplementary files.

## Supplementary Materials

Supplementary Materials Table S1 shows the clinical characteristics of GBS patients with and without folate deficiency.
