# Supplementary material for: Serum Folate Correlates with Severity of Guillain-Barré Syndrome and Predicts Disease Progression
Source: Biomed Res Int. 2018 Jun 14;2018:5703279. doi: 10.1155/2018/5703279 (PMC6022270; doi:10.1155/2018/5703279)
Supplement: Supplementary Materials — Table S1 shows the clinical characteristics of GBS patients with and without folate deficiency. [file 5703279.f1.docx]

Table S1 Characteristics of GBS patients with and without folate deficiency^a^

| Group | GBS with folate deficiency (n = 21) | GBS without folate deficiency (n = 91) | *p* |
| --- | --- | --- | --- |
| **Antecedent events** |  |  | 0.52 |
| Diarrhea | 23.8% (5/21) | 22.0% (20/91) |  |
| URTI | 19.0% (4/21) | 35.2% (32/91) |  |
| Both of the above | 4.8% (1/21) | 7.7% (7/91) |  |
| Gangliosides use | 14.3% (3/21) | 7.7% (7/91) |  |
| Others/Unknown | 38.1% (8/21) | 27.5% (25/91) |  |
| **Symptoms** |  |  |  |
| Numbness | 52.4% (11/21) | 49.5% (45/91) | 0.81 |
| Pain | 4.8% (1/21) | 22.0% (20/91) | 0.07 |
| Superficial sense deficit | 57.1% (12/21) | 50.5% (46/91) | 0.59 |
| Deep sensation deficit | 19.0% (4/21) | 9.9% (9/91) | 0.24 |
| Cranial nerves palsy | 33.3% (7/21) | 35.2% (31/91) | 0.87 |
| Dyspnea | 23.8% (5/21) | 18.7% (17/91) | 0.59 |
| **Respiratory failure** | 9.5% (2/21) | 8.8% (8/91) | 0.92 |
| **IVIG therapy** | 71.4% (15/21) | 75.8% (69/91) | 0.68 |
| **Laboratory results** |  |  |  |
| Serum folate, ng/mL | 3.07 (2.82-3.26) | 5.95 (4.44-8.38) | - |
| FPG, mmol/L (n = 103) | 6.09 (5.05-6.99) | 5.58 (4.99-6.51) | 0.41 |
| RBC count, 10^6^/µL (n=81) | 5.15 (4.27-5.52) | 4.69 (4.29-5.03) | 0.19 |
| Hemoglobin, g/L (n=81) | 149 (135-164) | 138 (127-152) | 0.12 |
| Mean Cell Volume, fL (n=81) | 85.7 (83.7-91.1) | 89.0 (86.3-91.6) | 0.20 |
| CSF Proteins, g/L (n = 89) | 1.08 (0.70-1.73) | 1.12 (0.65-1.65) | 0.75 |
| CSF IgG, mg/L (n = 72) | 141 (76.75-191.5) | 170 (63.43-372.5) | 0.56 |
| **Electrophysiology** (n = 99) |  |  | 0.76 |
| Demyelinating | 40.0% (6/15) | 50.0% (42/84) |  |
| Axonal | 20.0% (3/15) | 13.1% (11/84) |  |
| Mixed | 20.0% (3/15) | 10.7% (9/84) |  |
| Equivocal | 13.3% (2/15) | 15.5% (13/84) |  |
| Unremarkable | 6.7% (1/15) | 10.7% (9/84) |  |

Abbreviations: CSF, cerebrospinal fluid; FPG, fasting plasma glucose; IVIG, intravenous immunoglobulin; RBC, red blood cell; URTI, upper respiratory tract infection.

^a^Data are presented as percentage of patients unless otherwise indicated. All items are shown for 112 patients unless otherwise specified.
